# ^68^Ga-PSMA-11 PET/CT versus ^68^Ga-PSMA-11 PET/MRI for the detection of biochemically recurrent prostate cancer: a systematic review and meta-analysis

**DOI:** 10.3389/fonc.2023.1216894

**Published:** 2023-08-14

**Authors:** Ruizhe Huang, Yizhen Li, Haowen Wu, Boyi Liu, Xuanjun Zhang, Zhongxi Zhang

**Affiliations:** The First Clinical College, Changsha Medical University, Changsha, China

**Keywords:** PET/CT, PET/MRI, prostate cancer, biochemically recurrence, meta-analysis

## Abstract

**Purpose:**

Our aim was to conduct a meta-analysis and systematic review in order to compare the diagnostic efficacy of ^68^Ga-PSMA-11 PET/CT and ^68^Ga-PSMA-11 PET/MRI in patients with biochemically recurrent after radical prostatectomy and biochemically recurrent prostate cancers (BCR) after hybrid RT and RP.

**Methods:**

Up until February 2023, we searched PubMed, Embase, and Web of Science for pertinent papers. Studies examining the utility of ^68^Ga-PSMA-11 PET/CT or PET/MRI as a screening tool for biochemically recurrent prostate cancer were included. To measure heterogeneity, we employed the I^2^ statistic. In cases of substantial heterogeneity (I^2^ > 50%), we used the random effect model to produce a forest plot. In other cases, we utilized the fixed model. Furthermore, we assessed the quality of the studies included using the Quality Assessment of Diagnostic Performance Studies (QUADAS-2) method.

**Results:**

In total, 37 studies involving 8409 patients were examined. For ^68^Ga-PSMA-11 PET/CT and ^68^Ga-PSMA-11 PET/MRI, the combined total detection rate was 0.70 (95% CI: 0.65-0.75) and 0.71 (95% CI:0.67-0.75), respectively. ^68^Ga-PSMA-11 PET/CT and ^68^Ga-PSMA-11 PET/MRI did not substantially differ in terms of the overall detection rate for BCR *(P = 0.58)*. The detection rate was unaffected by the PSA values *(all P > 0.05)*.

**Conclusion:**

The diagnostic efficacy of ^68^Ga-PSMA-11 PET/CT appears to be equivalent to that of ^68^Ga-PSMA-11 PET/MRI in detecting biochemically recurrent prostate cancer. Nonetheless, it should be noted that not all studies have used pathological biopsies as the gold standard. Therefore, additional larger prospective studies are needed to address this issue.

**Systematic review registration:**

identifier CRD42023410039.

## 1 Introduction

One of the most prevalent diseases in the world, prostate cancer (PCa) has an annual incidence increase of 3% from 2014 to 2019 (1). Radiation therapy and radical surgery are the two most frequently used treatments for prostate cancer. A rise in prostate-specific antigen (PSA) levels following treatment, however, is a sign that over 30% of people may still experience disease recurrence (2, 3). In clinical practice, biochemical recurrence (BCR) of PCa is fairly typical. BCR is defined as an absolute rise in PSA level of 2 ng/ml over the lowest post-treatment PSA level following radiation therapy (RT) or a serum PSA level exceeding a threshold of 0.2 ng/ml twice after radical prostatectomy (RP) (4).

Imaging techniques are advised for individuals with biochemical recurrence who have serum prostate-specific antigen levels greater than 10 ng/mL or PSA doubling times shorter than 6 months (5). However, the ability of these traditional imaging techniques to diagnose aggressive lesions, bone involvement, and nodal metastases is restricted. It is essential to discover more sophisticated imaging techniques to detect the metastasis of the early BCR in order to increase diagnostic precision and select an appropriate treatment strategy.

EANM/SNMMI guidelines recently provided updated guidance and standards for the indication, acquisition, and interpretation of PSMA PET/CT for prostate cancer imaging (Fendler et al.). Currently, several guidelines highlight the superior accuracy of PSMA-ligand PET for staging primary disease (EAU, ESMO, NCCN) or consider additional value (ASCO) in this setting. PSMA-ligand PET/CT evaluation of BCR/BCP is recommended in documents produced by the EAU, ASCO, and NCCN (Fendler et al.). Moreover, evidence is growing in terms of PSMA-guided treatments, particularly metastases-directed therapy (Ceci et al., Rovera et al., Fendler et al., Phillips et al.) (6–9).

A type II membrane glycoprotein with 750 amino acids, prostate-specific membrane antigen(PSMA), is highly produced in prostate cancer cells (10). As a result, PSMA is thought to be a good candidate for PCa PET scanning. There are several radiopharmaceuticals that target PSMA, including ^68^Ga-PSMA-11, 18F-DCFPyL, and 18F-PSMA-1007 (11, 12).

Gallium-68 (^68^Ga)-labeled prostate-specific membrane antigen (PSMA-11), a new PET radiopharmaceutical, has recently gained attention as a promising imaging tool for the identification of recurrent prostate cancer. In patients with rising PSA levels, ^68^Ga-PSMA-11 PET has demonstrated great sensitivity and specificity for the detection of recurrent prostate cancer. However, uncertainty persists over the ideal imaging mode for ^68^Ga-PSMA-11 PET.

Despite systematic reviews or meta-analyses have evaluated the diagnostic efficacy of ^68^Ga-PSMA-11PET/CT and PET/MRI in earlier study, the amount of included article is insufficient (10, 13). This meta-analysis will enable more detailed and objective comparison of the diagnostic performance of ^68^Ga-PSMA-11 PET/CT and ^68^Ga-PSMA-11PET/MRI in detecting biochemical recurrent prostate. Our aim was to conduct a meta-analysis and systematic review in order to compare the diagnostic efficacy of ^68^Ga-PSMA-11 PET/CT and ^68^Ga-PSMA-11 PET/MRI in patients with biochemically recurrent prostate cancer in patient-based analysis.

## 2 Manuscript formatting

### 2.1 Materials and methods

This article was written according to Preferred Reporting Items for a Systematic Review and Meta-analysis of Diagnostic Test Accuracy (PRISMA-DTA) guidelines. Moreover, our registration number is CRD42023410039.

#### 2.1.1 Search strategy

The search strategy described below was used to perform a thorough search of the PubMed, Embase, and Web of Science databases until February 2023. (1) PET MRI OR PET MR OR positron emission tomography/magnetic resonance imaging OR PET CT OR positron emission tomography OR positron emission tomography/computed tomography; (2) regeneration OR recurrent OR relapse OR recrudescence; (3) prostate cancers OR prostate neoplasm OR prostate tumor OR prostatic tumor. For the reference list, we also go through the search and consider articles that may meet the inclusion criteria.

#### 2.1.2 Inclusion and exclusion criteria

Only study that fulfilled all of the following requirements were included: (1) articles evaluating the diagnostic performance of ^68^Ga-PSMA-11 PET/CT or ^68^Ga-PSMA-11 PET/MRI for biochemically recurrent prostate cancer in patient-based analysis; (2) number of patients ≥ 10; (3) retrospective or prospective studies; (4) English articles. The exclusion criteria were: (1) Irrelevant topic; (2) duplicated articles; (3) case reports, abstract, letters, review, or meta-analysis; (4) The full-text versions of the selected articles were screen to see if they fulfilled the inclusion criteria after the titles and abstracts of the articles were assessed in accordance with the inclusion and exclusion criteria. Disagreements among the researchers were resolved by consensus.

### 2.2 Quality assessment and data extraction

Two researchers separately evaluated the included studies’ quality using the Quality Assessment of Diagnostic Performance Studies (QUADAS-2) method. The applicability and bias risk of each study was assessed. Regarding bias risk and applicability, each study was given a rating of high, low, or unclear. A third reviewer was involved to resolve any possible conflicts. For the study, RevMan (version 5.4) was employed.

Two researchers independently extracted data for each of the included articles. The information that was extracted included the following: (1) the author, year of publication; (2) study characteristics, such as country, study design, analysis, and reference standard; (3) patient characteristics, such as number of patients, clinical indication, mean/median age, chemotherapy before PET; (4) technical characteristics, such as imaging test types, scanner modality, ligand dose, and time from injection to acquisition. When not explicitly mentioned, data were manually extracted from the literature, tables, and figures. We emailed the respective authors for more information when the paper lacked the information. Two researchers reached an accord to resolve their disagreements.

#### 2.2.1 Data synthesis and statistical analysis

Heterogeneity was assessed using the I^2^ statistic. A forest plot was constructed in the random-effect model if the significant heterogeneity was observed (I^2^ > 50%), otherwise, the fixed model would be applied. All of them used DerSimonian and Laird method. Proportions were transformed with the Freeman-Tukey double inverse sine transformation, and confidence intervals were calculated using the Jackson method. For the presence of heterogeneity (I^2^>50%), we used meta-regression and sensitivity analysis to find out the source of heterogeneity.

Publication bias was evaluated using Egger’s test. A statistically significant P value was two-tailed and with the threshold of 0.05. Statistical analyses were performed in R software environment for statistical computing and graphics version 4.2.2

### 2.3 Results

#### 2.3.1 Literature search and study selection

**Figure 1:**
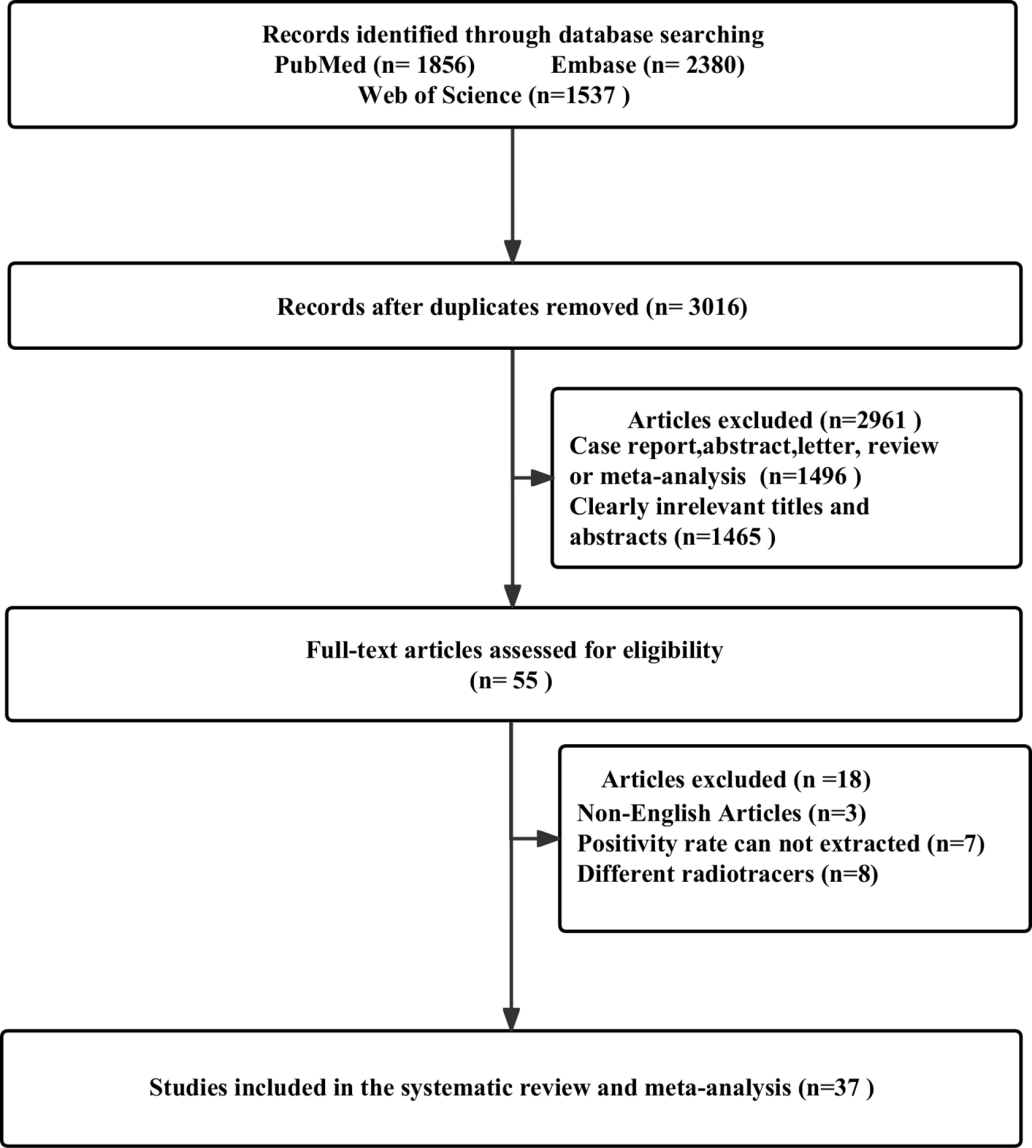
The flow chart for the PRISMA study selection procedure.

After removing 2757 duplicate studies from the primary search, 3016 studies were identified out of the 5773 articles that were initially found. Based on the study’s title or abstract, 1465 papers were disqualified. A total of 1496 investigations were collectively omitted from case reports, abstracts, letters, reviews, or meta-analyses. There were still 55 studies for full-text screening, and another 18 were disqualified due to the following reasons: non-English studies (n = 3); cannot extract positivity rate data (n = 7); and different radiotracers (n = 8). 37 studies that satisfied the criteria for the meta-analysis were finally included, including 25 articles for ^68^Ga-PSMA-11 PET/CT (14–38) and 13 articles for ^68^Ga-PSMA-11 PET/MRI (4, 26, 39–49). One of the studies included not only ^68^Ga-PSMA-11 PET/CT but also ^68^Ga-PSMA-11 PET/MRI. **Figure 1** illustrates the PRISMA flow chart of the study selection procedure.

#### 2.3.2 Study description and quality assessment study description and quality assessment

**Figure 2:**
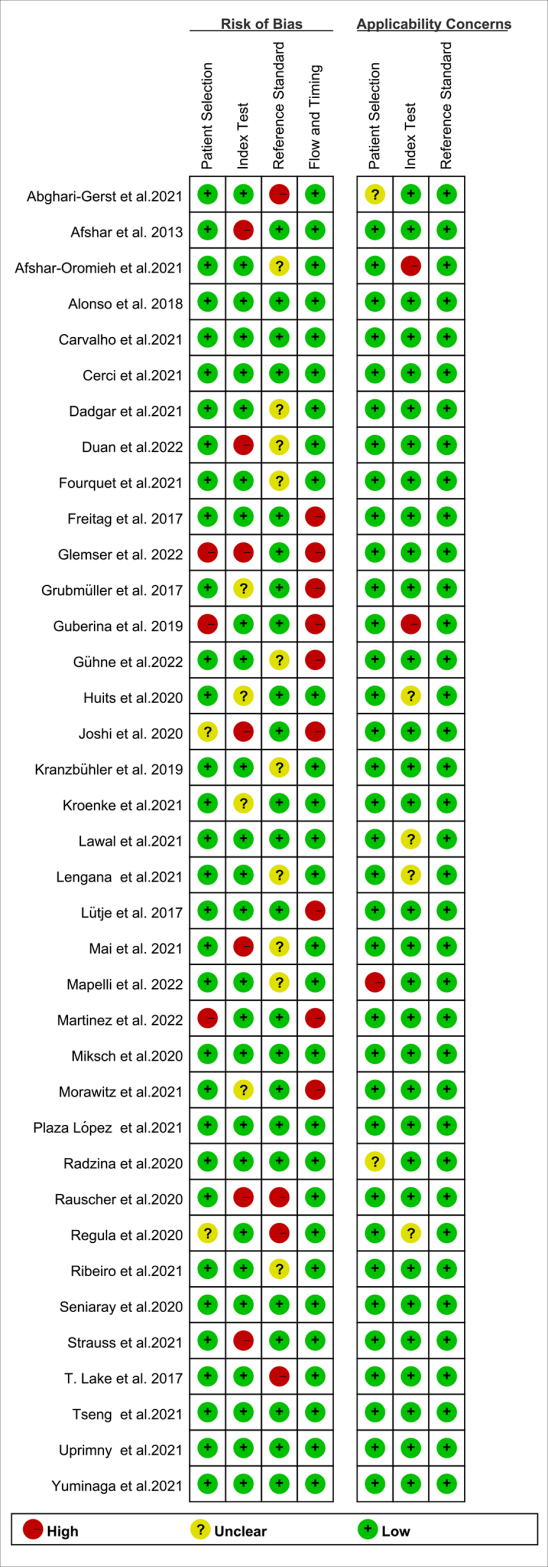
Graph of risk of bias and applicability of all eligible studies based on QUADAS-2 tool.

The study and patient characteristics from the 37 studies covering 8409 patients were listed in **Table 1**. **Tables 2**, **3** showed the technical the parts. Additionally, using the Quality Assessment of Diagnostic Accuracy Studies (QUADAS-2) tool, a quality assessment of the relevant studies was conducted. The quality evaluation chart reveals that flow and timing are the key areas where there is a high risk of bias (**Figure 2**). This is due to the fact that most studies did not analyze all of the enrolled patients, which caused this issue. Overall, the risk of bias of the included articles was satisfactory.

**Table 1:** Characteristics of the studies and patients.

Author	Year	Types of imaging tests	Study characteristics					Patient characteristics	
			Country	Study design	Analysis	No. of patients	PSA level prior to PET (ng/ml)	Mean/Median age	Previous treatment
Gühne et al.	2022	PET/CT	Germany	Retro	PB	83	Median = 1.3	Median = 70	Mixed
Duan et al. (15)	2022	PET/CT	USA	Pro	PB	58	NA	NA	Mixed
Uprimny et al. (16)	2021	PET/CT	Austria	Retro	PB	440	NA	NA	Mixed
Lengana et al. (17)	2021	PET/CT	South Africa	Pro	PB	21	Mean = 2.6	Mean = 68.6	Mixed
Plaza López et al. (18)	2021	PET/CT	Spain	Retro	PB	14	Mean = 1.8	Mean = 71.1	Mixed
Yuminaga et al. (19)	2021	PET/CT	Australia	Pro	PB	384	Median = 0.5	Median = 69.5	RP
Tseng et al. (20)	2021	PET/CT	China	Pro	PB	34	Median = 0.5	Median = 67	RP
Strauss et al. (21)	2021	PET/CT	Germany	Retro	PB	142	Median = 2.3	NA	RP
Ribeiro et al. (22)	2021	PET/CT	Brazil	Retro	PB	57	NA	Median = 69	NA
Morawitz et al. (23)	2021	PET/CT	Germany	Retro	PB	36	Median = 1.5	Median = 71	RP
Lawal et al. (24)	2021	PET/CT	South Africa	Retro	PB	247	Median = 2.7	Mean = 65.7	Mixed
Kroenke et al. (25)	2021	PET/CT	Germany	Retro	PB	127	Median = 0.7	Median = 69.0	RP
Jentjens et al.	2021	PET/CT	Belgium	Pro	PB	34	Median = 0.8	Median = 67.5	Mixed
Fourquet et al. (27)	2021	PET/CT	France	Retro	PB	294	NA	Mean = 68.0	RP
Dadgar et al. (28)	2021	PET/CT	Iran	Retro	PB	19	Median = 1.7	Median = 72.0	Mixed
Cerci et al.	2021	PET/CT	Brazil	Pro	PB	1004	Mean = 1.6	Mean = 67.3	Mixed
Carvalho et al. (30)	2021	PET/CT	Portugal	Pro	PB	70	NA	NA	Mixed
Afshar-Oromieh et al. (31)	2021	PET/CT	Germany	Retro	PB	2533	NA	Median = 68	RP
Abghari-Gerst et al.	2021	PET/CT	USA	Pro	PB	1539	median:7	Mean = 67.3	Mixed
Seniaray et al. (33)	2020	PET/CT	India	Retro	PB	170	NA	NA	Mixed
Regula et al. (34)	2020	PET/CT	Sweden	Pro	PB	30	Median = 5	Median = 70	Mixed
Rauscher et al. (35)	2020	PET/CT	Germany	Retro	PB	102	Median = 0.9	Median = 69	RP
Radzina et al. (36)	2020	PET/CT	Latvia	Pro	PB	32	Median = 1.1	Mean = 63	Mixed
Miksch et al. (37)	2020	PET/CT	Germany	Retro	PB	116	Mean = 0.2	Mean = 67.6	RP
Huits et al. (38)	2020	PET/CT	Netherlands	Retro	PB	100	Median = 0.5	Mean = 65	RP
Glemser et al. (47)	2022	PET/MRI	Germany	Retro	PB	53	median:1.6	mean:67.7	mixed
Afshar et al.	2013	PET/MRI	Germany	Pro	PB	20	median:2.62	mean:69.6	mixed
Grubmüller et al.	2017	PET/MRI	Austria	Retro	PB	71	median:1.04	NA	RP
Guberina et al.	2019	PET/MRI	Germany	Retro	PB	93	median:1.64	NA	RP
Mai et al.	2021	PET/MRI	Belgium	Pro	PB	20	median:0.79	mean:67.5	mixed
Joshi et al. (43)	2020	PET/MRI	Australia	Pro	PB	21	median:0.69	median:68	mixed
T. Lake et al. (41)	2017	PET/MRI	USA	Pro	PB	55	mean:7.9	mean:68.3	mixed
Kranzbühler et al.	2019	PET/MRI	Switzerland	Retro	PB	66	median:0.23	NA	mixed
Lütje et al. (40)	2017	PET/MRI	Germany	Retro	PB	25	mean3.9	mean:70.5	RP
Mapelli et al. (39)	2022	PET/MRI	Italy	Pro	PB	35	mean:1.88	mean:70	mixed
Martinez et al. (4)	2022	PET/MRI	USA	Pro	PB	109	mean:5.56	mean:69	mixed
Alonso et al. (48)	2018	PET/MRI	India	Pro	PB	36	median:3.3	mean:64.7	mixed
Freitag et al. (49)	2017	PET/MRI	Germany	Retro	PB	119	median:1.70	NA	RP

PB, patient-based; Pro, prospective; Retro, retrospective; NA, not available.

**Table 2:** Technical aspects of included ^68^Ga-PSMA-11 PET/MRI studies.

Author	Year	Scanner Modality(PET/MRI)	Ligand dose	Time from injection to acquisition	Image analysis
Glemser et al. (47)	2022	NA	71-287MBq	170min for PET/MRI	quantitative
Afshar et al.	2013	Siemens Biograph	76-259 MBq	90min for PET/MRI	quantitative
Grubmüller et al.	2017	Siemens Biograph Mmr	NA	NA	qualitative
Guberina et al.	2019	Siemens Biograph Mmr	66–167 MBq	167min for PET/MRI	quantitative
Mai et al.	2021	GE Healthcare	NA	NA	quantitative
Joshi et al. (43)	2020	NA	150MBq	45-60min for PET/MRI	quantitative
T. Lake et al. (41)	2017	GE SIGNA PET/MR	201.5 ± 52.9 MBq	65min for PET/MRI	qualitative
Kranzbühler et al.	2019	GE SIGNA PET/MR	130 ± 16 MBq	NA	quantitative
Lütje et al. (40)	2017	Siemens Healthcare	118 ± 23 MBq	175 ± 45 min for PET/MRI	quantitative
Mapelli et al. (39)	2022	SIGNA PET/MRI	129-288MBq	60min for PET/MRI	quantitative
Martinez et al. (4)	2022	Siemens Biograph mMR	148MBq	90min for PET/MRI	quantitative
Alonso et al. (48)	2018	GE Discovery	2.0 MBq/kg	NA	quantitative
Freitag et al. (49)	2017	Siemens Biograph mMR	202 ± 69 MBq	70min for PET/MRI	quantitative

**Table 3:** Technical aspects of included ^68^Ga-PSMA-11 PET/CT studies.

Author	Year	Scanner Modality(PET/CT)	Ligand dose	Time from injection to acquisition	Image analysis
Gühne et al.	2022	Siemens Healthineers	243.2 ± 35.8MBq	71min for PET/CT	quantitative
Duan et al. (15)	2022	GE Healthcare	146.5 ± 16.7MBq	86.8 ± 11.5min for PET/CT	quantitative
Uprimny et al. (16)	2021	GE Healthcare	95.0–216.0 MBq	67min for PET/CT	quantitative
Lengana et al. (17)	2021	Siemens Biograph 40	NA	60min for PET/CT	qualitative
Plaza López et al. (18)	2021	GE HealthCare	2.2 MBq/kg	60min for PET/CT	qualitative
Yuminaga et al. (19)	2021	Philips GEMINI TOF	150-300 MBq	50-70min for PET/CT	qualitative
Tseng et al. (20)	2021	Siemens Healthineers	88.4-182.8 MBq	60min for PET/CT	qualitative
Strauss et al. (21)	2021	Siemens Biograph	193.6 ± 62.87MBq	80-90min for PET/CT	quantitative
Ribeiro et al. (22)	2021	Philips Health	NA	60min for PET/CT	qualitative
Morawitz et al. (23)	2021	Siemens Healthineers	182 ± 45 MBq	NA	quantitative
Lawal et al. (24)	2021	Siemens Medical Solution	2MBq/kg	60min for PET/CT	qualitative
Kroenke et al. (25)	2021	Siemens Medical Solutions	51-248 MBq	42-116min for PET/CT	quantitative
Jentjens et al.	2021	Siemens Healthineers	1.8MBq/kg	60min for PET/CT	quantitative
Fourquet et al. (27)	2021	Philips Medical Systems	1.2MBq/kg	60-90min for PET/CT	quantitative
Dadgar et al. (28)	2021	Siemens	126-187 MBq	60min for PET/CT	quantitative
Cerci et al.	2021	NA	2MBq/kg	60-90min for PET/CT	qualitative
Carvalho et al. (30)	2021	Siemens Biography	2MBq/kg	60min for PET/CT	quantitative
Afshar-Oromieh et al. (31)	2021	NA	52–480 MBq	NA	quantitative
Seniaray et al. (33)	2020	NA	132–222 MBq	45 ± 15min for PET/CT	qualitative
Regula et al. (34)	2020	GE Healthcare	1.3–2.9 MBq/kg	60-78min for PET/CT	quantitative
Rauscher et al. (35)	2020	Biograph-Mct	94–232 MBq	41-85min for PET/CT	quantitative
Radzina et al. (36)	2020	Gemini TF64	1.8-2.2 MBq/kg	51-81min for PET/CT	quantitative
Miksch et al. (37)	2020	Siemens Biograph-Mct	162.7 ± 22.3 MBq	64.4 ± 12.2min for PET/CT	qualitative
Huits et al. (38)	2020	Philips Ingenuity	2.0 MBq/kg	60min for PET/CT	qualitative
Abghari-Gerst et al.	2021	GE Discovery	NA	61min for PET/CT	quantitative

#### 2.3.3 Diagnostic performance of ^68^Ga-PSMA-11 PET/CT and PET/MRI for biochemically recurrent prostate cancer

**Figure 3:**
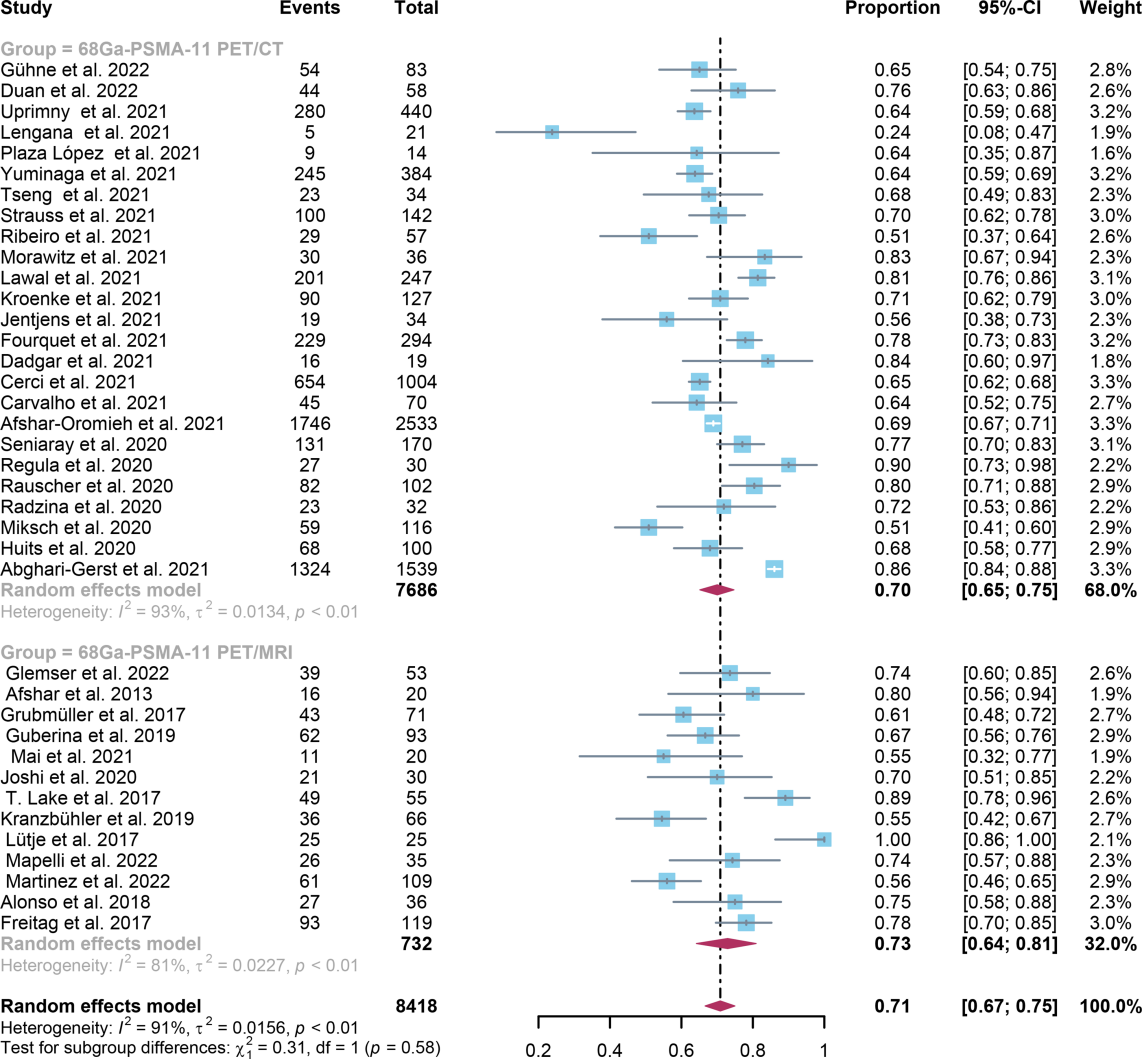
^68^Ga-PSMA-11 PET/CT and ^68^Ga-PSMA-11 PET/MRI forest plots for biochemically recurrent prostate cancer. In each study, positive results were represented by squares, and the 95% confidence interval was shown by horizontal bars.

In comparison to ^68^Ga-PSMA-11 PET/CT, which had a positivity rate of 0.70 (95% Cl: 0.65-0.75), ^68^Ga-PSMA-11 PET/MRI had a positivity rate of 0.71 (95% Cl: 0.67-0.75). The analysis included 8409 patients from 37 studies. There was no statistically significant difference in the overall detection rate between ^68^Ga-PSMA-11 PET/CT and ^68^Ga-PSMA-11 PET/MRI (*P=0.58)* (**Figure 3**).

Regarding the pooled overall detection rate of ^68^Ga-PSMA-11 PET/CT and ^68^Ga-PSMA-11 PET/MRI for BCR, the I^2^ was 93% and 81%, respectively. For ^68^Ga-PSMA-11 PET/CT, the subgroup analysis and meta-regression analysis showed that the data analysis (qualitative vs. quantitative) was the possible cause of heterogeneity, while the study design (The number of patients: Greater than 56 vs. less than or equal to 56) was identified as the potential cause of heterogeneity for the ^68^Ga-PSMA-11 PET/MRI studies **Tables 4**, **5**. There were no potential sources of heterogeneity found by the sensitivity analysis. The result revealed only slight variations in the data, with values ranging from 0.70 to 0.74 for the ^68^Ga-PSMA-11 PET/MRI and from 0.69 to 0.71 for the ^68^Ga-PSMA-11 PET/CT. Overall, the detection rates remained consistent and stable after sensitivity analysis. (**Supplementary Tables 1, 2**).

**Table 4:** Subgroup analysis and meta-regression analysis of diagnostic performance of ^68^Ga-PSMA-11 PET/MRI.

Covariate/Subgroup	Studies, n	Positivity rate (95%CI)	P-value
Study design			0.82
Prospective	7	0.74(0.58-0.88)	
Retrospective	6	0.72(0.61-0.82)	
Treatment			0.35
RP	4	0.73(0.64-0.81)	
Mixed	9	0.70(0.61-0.78)	
The number of patients			0.03
>56	5	0.64(0.55-0.73)	
≤56	8	0.79(0.68-0.89)	
Image analysis			0.72
Qualitative	2	0.73(0.64-0.81)	
Quantitative	11	0.72(0.63-0.81)	

Table 5Subgroup analysis and meta-regression analysis of diagnostic performance of ^68^Ga-PSMA-11 PET/CT.

**Table:** 

Covariate/Subgroup	Studies, n	Positivity rate (95%CI)	P-value
Study design			0.66
Prospective	10	0.70(0.65-0.75)	
Retrospective	15	0.71(0.66-0.76)	
Treatment			0.90
RP	10	0.71(0.66-0.75)	
Mixed	14	0.71(0.63-0.78)	
The number of patients			0.93
>307	20	0.70(0.64-0.76)	
≤307	5	0.70(0.61-0.79)	
Image analysis			0.03
Qualitative	10	0.70(0.65-0.75)	
Quantitative	15	0.74(0.69-0.79)

**Table:** 

8.7 Positivity analysis of overall detection rate for ^68^Ga-PSMA-11 PET/MRI		
8.8	8.9 ^68^Ga-PSMA-11 PET/MRI	
	8.10 Positivity rate (95% CI)	8.11 I^2^
8.12 Omitting Glemser et al.	8.13 0.73 [0.63,0.82]	8.14 82.40%
8.15 Omitting Afshar et al.	8.16 0.72 [0.63,0.81]	8.17 82.30%
8.18 Omitting Grubmüller et al.	8.19 0.74 [0.65, 0.82]	8.20 81.50%
8.21 Omitting Guberina et al.	8.22 0.74 [0.64, 0.82]	8.23 82.30%
8.24 Omitting Mai et al.	8.25 0.74 [0.65,0.82]	8.26 81.90%
8.27 Omitting Joshi et al.	8.28 0.73 [0.64,0.82]	8.29 82.50%
8.30 Omitting T. Lake et al.	8.31 0.71 [0.62, 0.79]	8.32 78.10%
8.33 Omitting Kranzbühler et al.	8.34 0.74 [0.66,0.82]	8.35 80.00%
8.36 Omitting Lütje et al.	8.37 0.70 [0.63, 0.76]	8.38 71.60%
8.39 Omitting Mapelli et al.	8.40 0.73 [0.63, 0.82]	8.41 82.40%
8.42 Omitting Martinez et al.	8.43 0.74 [0.66, 0.83]	8.44 78.60%
8.45 Omitting Alonso et al.	8.46 0.73 [0.63, 0.81]	8.47 82.40%
8.48 Omitting Freitag et al.	8.49 0.72 [0.63, 0.81]	8.50 81.20%

According to the funnel plot and Egger’s test, both the ^68^Ga-PSMA-11 PET/CT *(P = 0.39)* and the ^68^Ga-PSMA-11 PET/MRI *(P = 0.28)* showed no sign of publication bias (**Supplementary Tables 1 and 2**).

#### 2.3.4 BCR positivity rate for ^68^Ga-PSMA-11 PET/CT and ^68^Ga-PSMA-11 PET/MRI according to the different PSA subgroups

**Figure 4:**
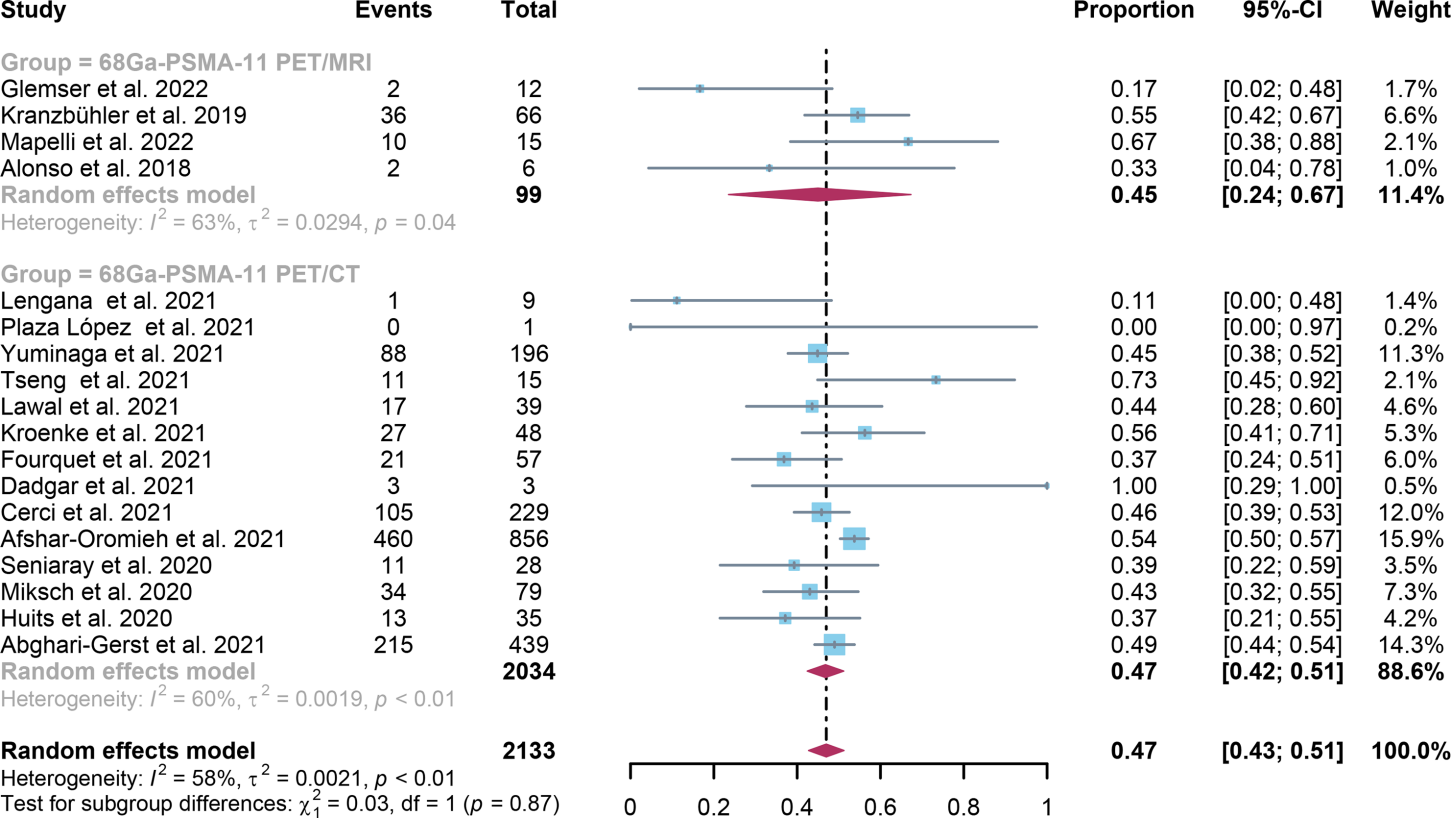
Forest plot of ^68^Ga-PSMA-11 PET/CT and ^68^Ga-PSMA-11 PET/MRI detection rates in patients with PSA<0.5.

**Figure 5:**
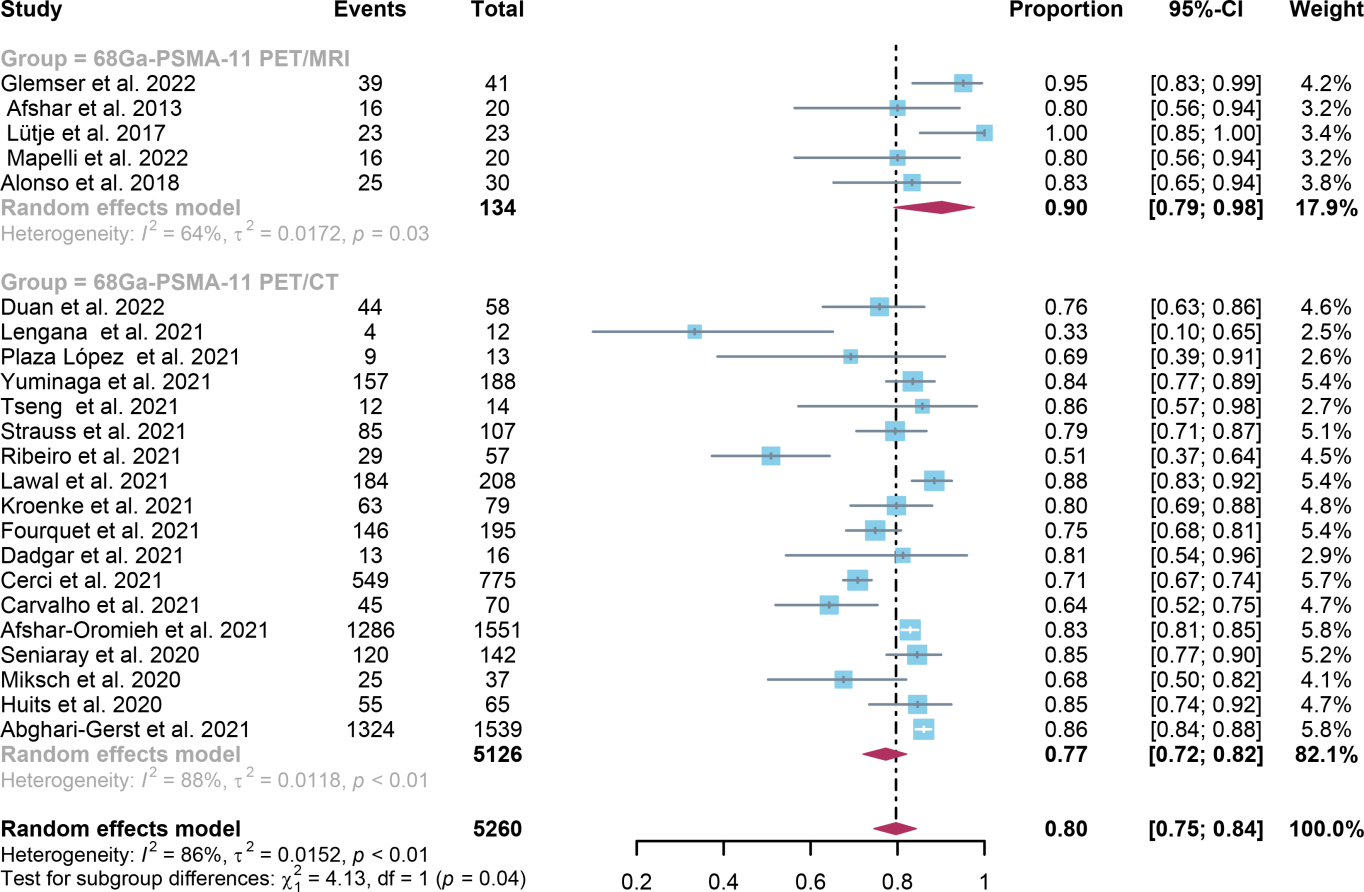
Forest plot of ^68^Ga-PSMA-11 PET/CT and ^68^Ga-PSMA-11 PET/MRI detection rates in patients with PSA>0.5.

**Figure 6:**
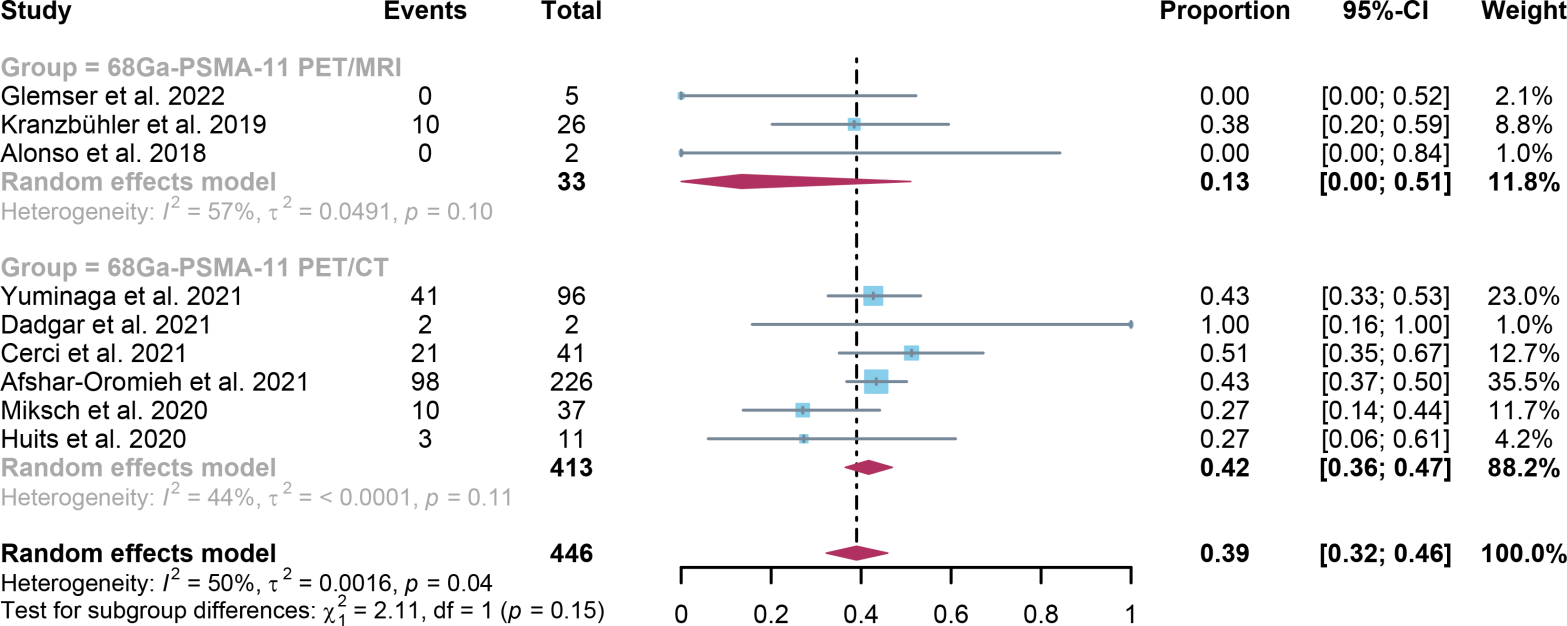
Forest plot of ^68^Ga-PSMA-11 PET/CT and ^68^Ga-PSMA-11 PET/MRI detection rates in patients with PSA≤ 0.2.

**Figure 7:**
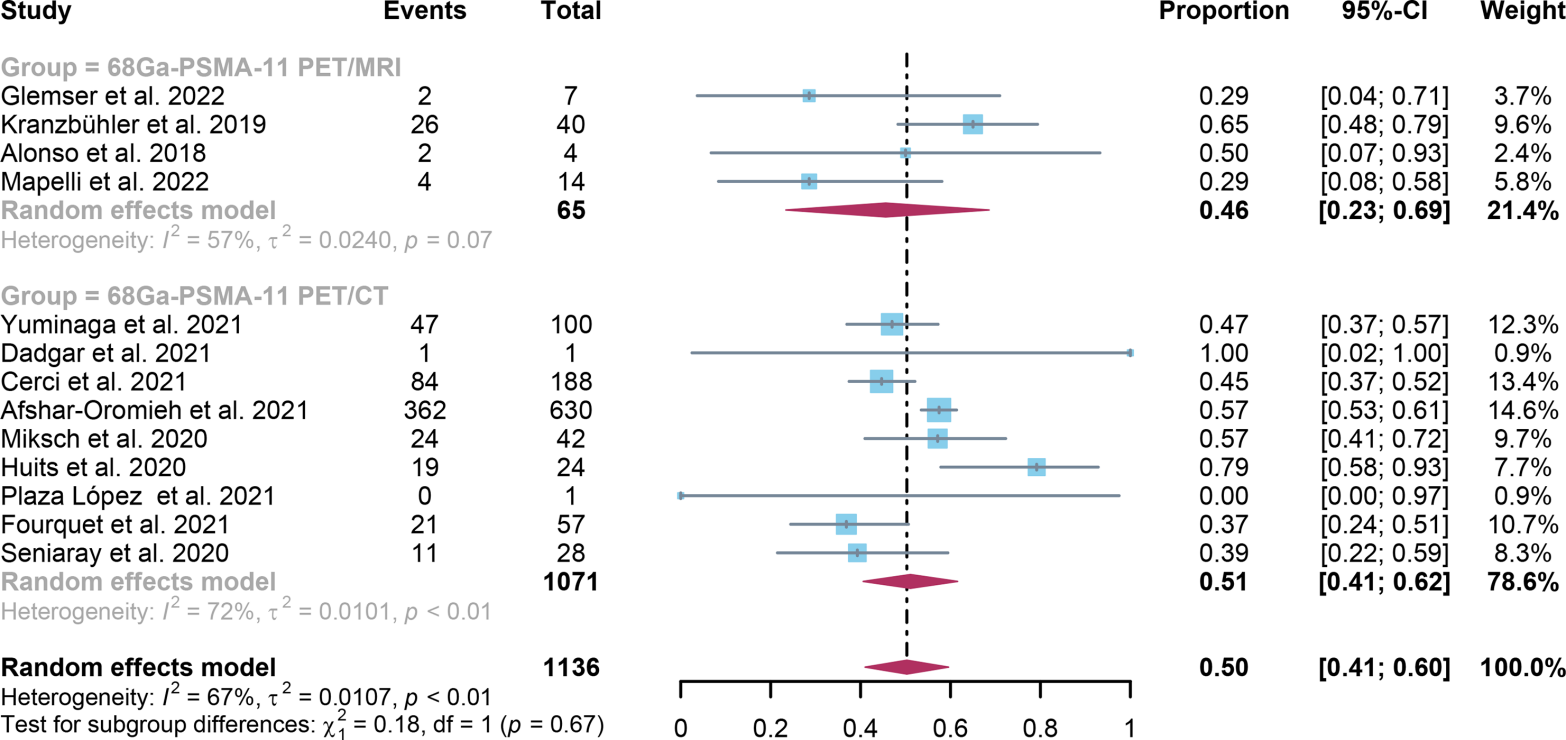
Forest plot of ^68^Ga-PSMA-11 PET/CT and ^68^Ga-PSMA-11 PET/MRI detection rates in patients with 0.2<PSA<0.5.

**Figure 8:**
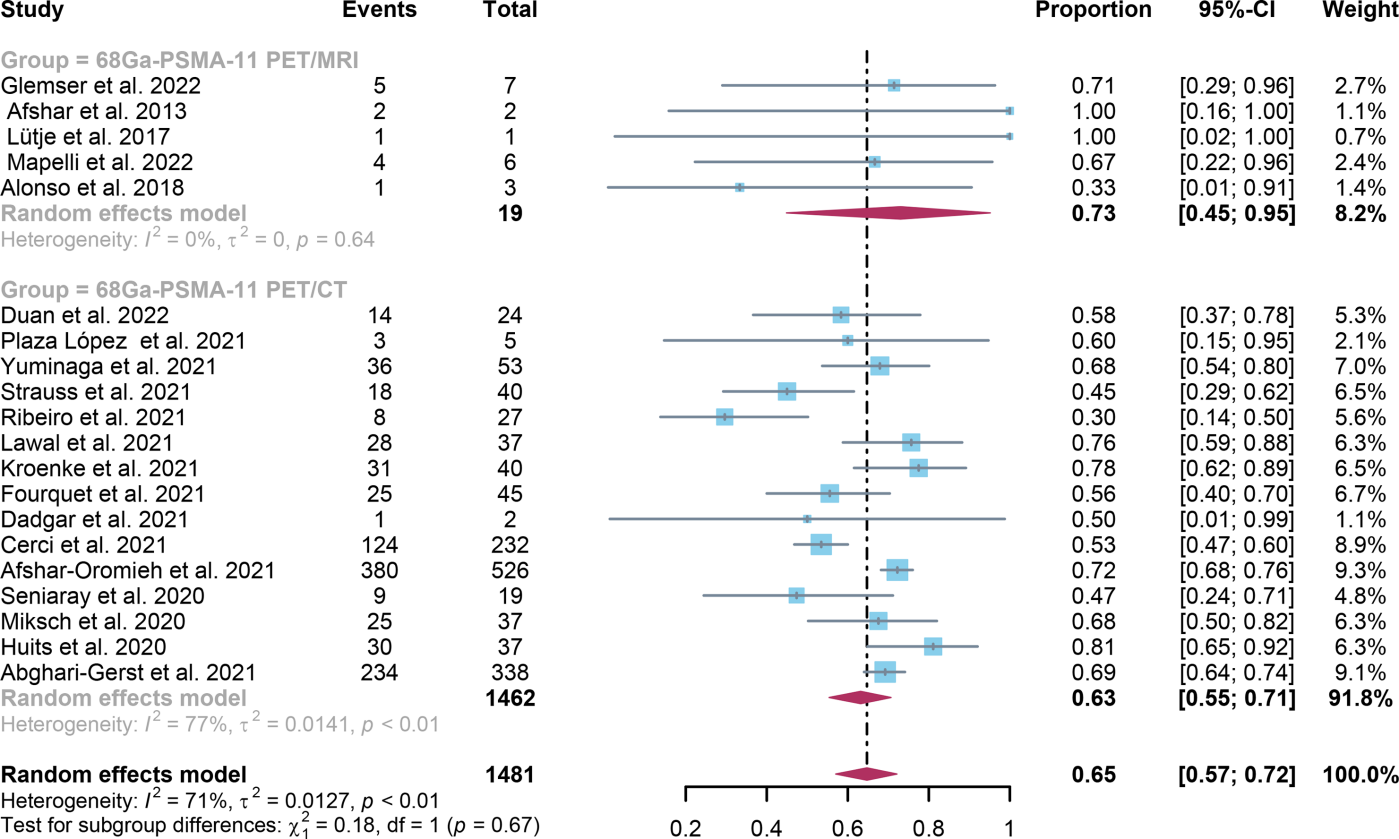
Forest plot of ^68^Ga-PSMA-11 PET/CT and ^68^Ga-PSMA-11 PET/MRI detection rates in patients with 0.5≤PSA<1.0.

**Figure 9:**
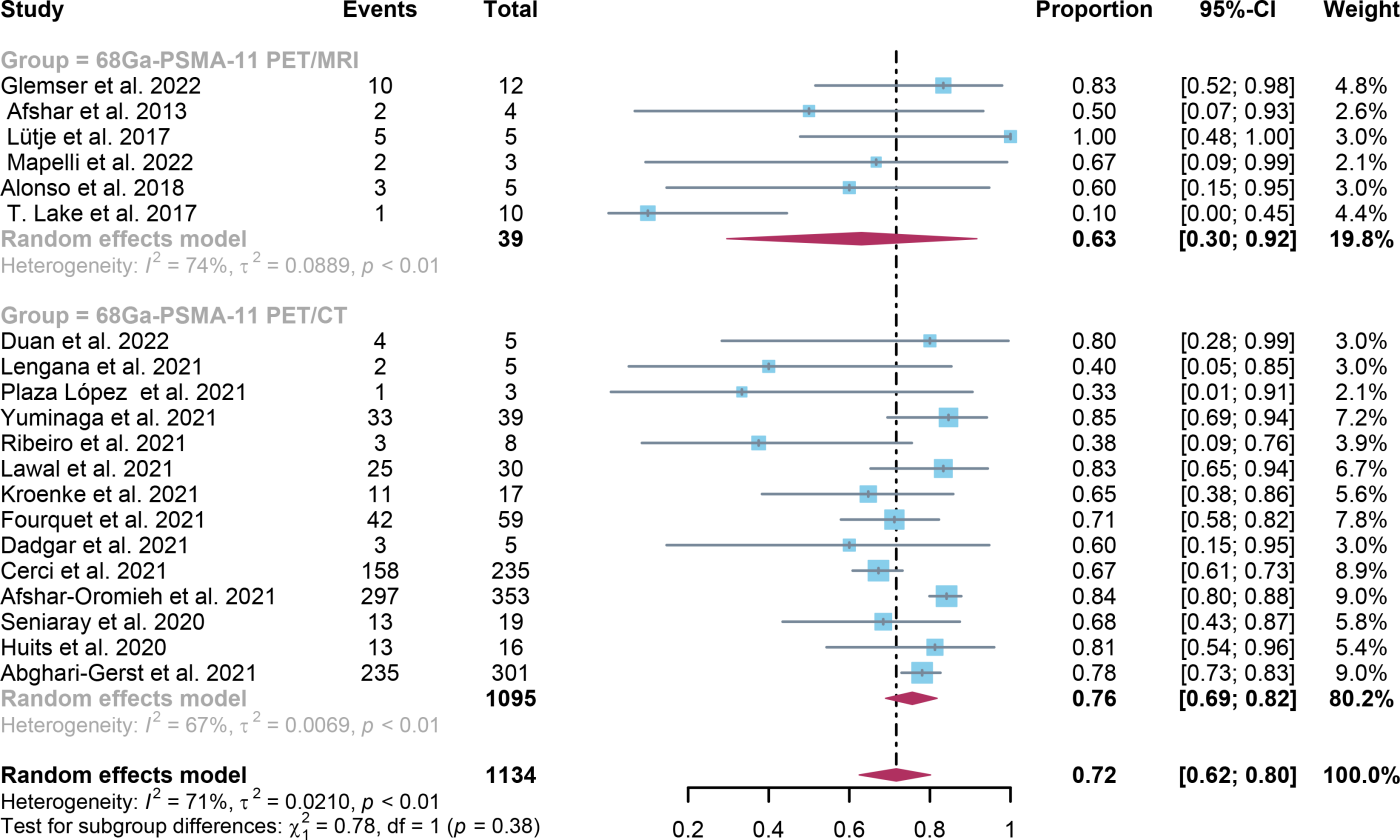
Forest plot of ^68^Ga-PSMA-11 PET/CT and ^68^Ga-PSMA-11 PET/MRI detection rates in patients with 1.0≤PSA<2.0.

**Figure 10:**
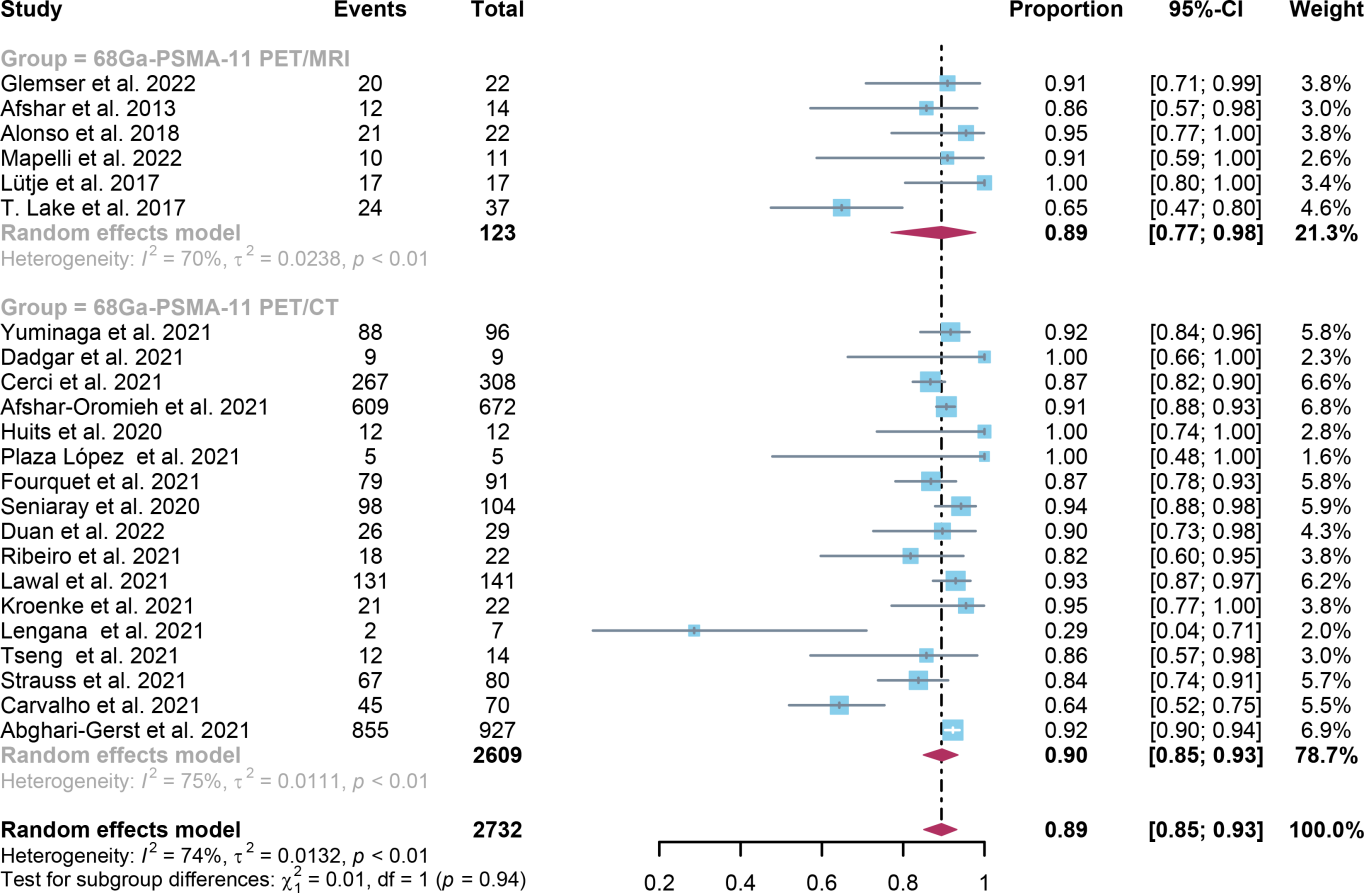
Forest plot of ^68^Ga-PSMA-11 PET/CT and ^68^Ga-PSMA-11 PET/MRI detection rates in patients with PSA≥2.0.

For ^68^Ga-PSMA-11 PET/CT and ^68^Ga-PSMA-11 PET/MRI, the detection rates were 0.47 (95% CI: 0.42-0.51) and 0.45 (95% CI: 0.24-0.67) for PSA levels <0.5 ng/ml, and when PSA levels were > 0.5 ng/ml, the detection rates were 0.77 (95% CI: 0.72-0.82) and 0.90 (95% CI: 0.79-0.98); Detection rates at PSA levels <0.2 ng/ml were 0.42 (95% CI: 0.36-0.47) and 0.13 (95% CI: 0.00-0.51); For PSA levels 0.2-0.5 ng/ml, the detection rates were 0.51 (95% CI: 0.41-0.62) and 0.46 (95% CI: 0.23-0.69); For PSA levels 0.5-1.0 ng/mL, detection rates were 0.63 (95% CI: 0.55-0.71) and 0.73 (95% CI: 0.45-0.95); For PSA values 1.0-2.0 ng/mL, the detection rates were 0.76 (95% CI: 0.69-0.82) and 0.63 (95% CI: 0.30-0.92); the detection rates for PSA levels > 2.0 ng/ml were 0.90 (95% CI: 0.85-0.93) and 0.89 (95% CI: 0.77-0.98). The only significant difference ^68^Ga-PSMA-11 PET/CT and ^68^Ga-PSMA-11 PET/MRI was at PSA levels > 0.5 ng/ml *(P=0.04)* (**Figures 4**–**10**).

### 2.4 Discussion

According to previous studies, PET/MRI with PSMA imaging agent has a slightly higher diagnostic performance than PET/CT for local recurrence and lymph node recurrence (10, 44, 46, 49). However, according to Huo et al. and Glemser et al., there is no significant difference between the overall detection rates of the two imaging modalities (13, 47). Thus controversy remains regarding the diagnostic performance of both imaging modalities for biochemical recurrent prostate cancer. The aim of this study was to quantitatively compare the diagnostic performance of the two diagnostic modalities for biochemical recurrent prostate cancer.

In the present study, the capability of two imaging modalities to identify BCR was comprehensively reviewed and assessed. The detection rates in patient-based analysis for ^68^Ga-PSMA-11 PET/CT and ^68^Ga-PSMA-11 PET/MRI were 0.70 (95% Cl: 0.65; 0.75) and 0.73 (95% Cl: 0.64; 0.81) accordingly. Between these two imaging modalities, there was no significant difference *(P=0.58)*. A significant difference between these two imaging modalities for PSA levels aspect only existed when PSA was higher than 0.5, according to the study *(P=0.04) *(**Figure 7**).

PET/MRI provides metabolic, anatomical, and functional information in a single modality by combining the strengths of PET and MRI. While MRI offers precise anatomical and functional information through techniques like perfusion and diffusion-weighted imaging, PSMA-11 PET provides metabolic information by detecting PSMA expression in prostate cancer cells. With the use of this extensive information, clinicians can make a more thorough evaluation of the kind and severity of prostate cancer. PET/MRI detection rates may be superior to PET/CT for PSA levels higher than 0.5 due to the fact that PET/MRI provides more precise and detailed anatomical data, particularly in terms of soft tissue contrast (48). In order to more precisely localize probable tumor lesions, PSMA-11 PET/MRI can provide detailed anatomical information on the prostate region, including its shape, location, and size (36).

In addition, compared to PET/CT, PET/MRI often has lower radiation doses, which may be advantageous for younger patients or those who need numerous follow-up exams, lowering the risk of radiation exposure. It’s important to remember that clinical practices may vary between different medical facilities, even though PSMA-11 PET/MRI may be advantageous when PSA is greater than 0.5. It is important to stress that this is only an observation or trend and does not always mean that PSMA-11 PET/MRI is always preferable than PSMA-11 PET/CT.

Compared to previous meta-analyses (13), the current meta-study found that ^68^Ga-PSMA-11 PET CT and ^68^Ga-PSM A-11 PET-MRI had similar results in terms of diagnostic performance and detection rates for the detection of biochemically recurrent prostate cancer. This shows that for the same detection performance, PET/CT is more cost-effective. These findings are consistent with previous meta-analyses. The main disadvantage of the previous meta-analysis is the small sample size, while the main advantage of the meta-analysis in this article is the large sample size(including 37 studies). However, due to the recent development of ^68^Ga-PSMA-11 PET/MRI, there is limited study in this field and a scarcity of comparable evidence available. Future head-to-head studies that systematically assesses both modalities might produce novel findings.

The findings of the meta-analysis contrasting ^68^Ga-PSMA-11 PET/CT and ^68^Ga-PSMA-11 PET/MRI for the identification of biochemically recurring prostate cancer can have significant repercussions for future study in the field as well as for policy and practice. To make the best use of various imaging modalities in clinical practice, these findings can inspire future study paths, help decision-making, and enhance patient management. The diagnosis of PSMA-PET has a significant impact on the management of recurrent patients, allowing clinicians to select better treatment options to treat them, such as the treatment of recurrent M1a prostate cancer, the MDT approach of targeting PSMA-positive lesions according to the pattern of recurrence (sLND, SBRT, combination of sLND and SBRT). Based on the PSMA-PET method, these treatments were chosen (50–53).

Both ^68^Ga-PSMA-11 PET/CT and ^68^Ga-PSMA-11 PET/MRI demonstrated high heterogeneity in terms of overall detection rates. In an attempt to find out the source of this heterogeneity, we conducted sensitivity analysis and meta-regression. Our findings showed that for PET/CT, the primary cause of heterogeneity was image analysis *(P=0.03)*. On the other hand, for PET/MRI, the primary cause of heterogeneity appeared to be the number of patients involved in the studies *(P=0.03)*. The sensitivity analysis did not identify any potential sources of heterogeneity.

It is also important to note the limitations of our meta-analysis. First of all, the gold standard for pathology was not available for all of the patients. Secondly, many of the included studies were retrospective studies, further lager prospective studies are needed. Finally, the included study used various protocols, such as different methods for administering contrast agents, different contrast procedures, and varied standards for interpretation, which may cause heterogeneity.

### 2.5 Conclusion

The diagnostic efficacy of ^68^Ga-PSMA-11 PET/CT appears to be equivalent to that of ^68^Ga-PSMA-11 PET/MRI in detecting biochemically recurrent prostate cancer. Nonetheless, it should be noted that not all studies have used pathological biopsies as the gold standard. Therefore, additional larger head-to-head prospective studies are needed to address this issue.

## Data availability statement

The original contributions presented in the study are included in the article/**Supplementary Material**. Further inquiries can be directed to the corresponding author.

## Author contributions

The study was conceptualized and designed by RH and ZZ, and it was verified using information gathered and examined by YL, HW, BL, and XZ. The manuscript was written by RH. The article’s submission was reviewed and approved by all of the writers.
