# Supplementary material for: 68Ga-PSMA-11 PET/CT versus 68Ga-PSMA-11 PET/MRI for the detection of biochemically recurrent prostate cancer: a systematic review and meta-analysis
Source: Front Oncol. 2023 Aug 14;13:1216894. doi: 10.3389/fonc.2023.1216894 (PMC10461474; doi:10.3389/fonc.2023.1216894)
Supplement: Supplementary file 1 [file DataSheet_1.pdf]

Supplementary Figure 1 Publication bias was evaluated for 68Ga-PSMA-11 PET/CT using Deek's funnel plot. A significance level of P<0.05 was used to define statistical significance.

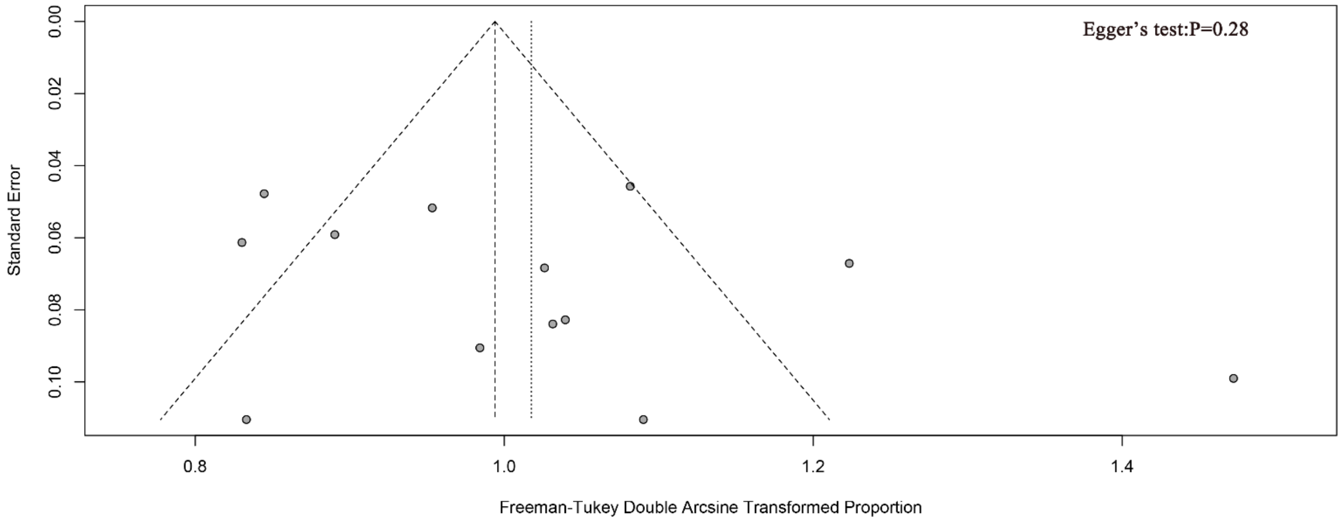

Supplementary Figure 2 Publication bias was evaluated for 68Ga-PSMA-11 PET/MRI using Deek's funnel plot. A significance level of P<0.05 was used to define statistical significance.

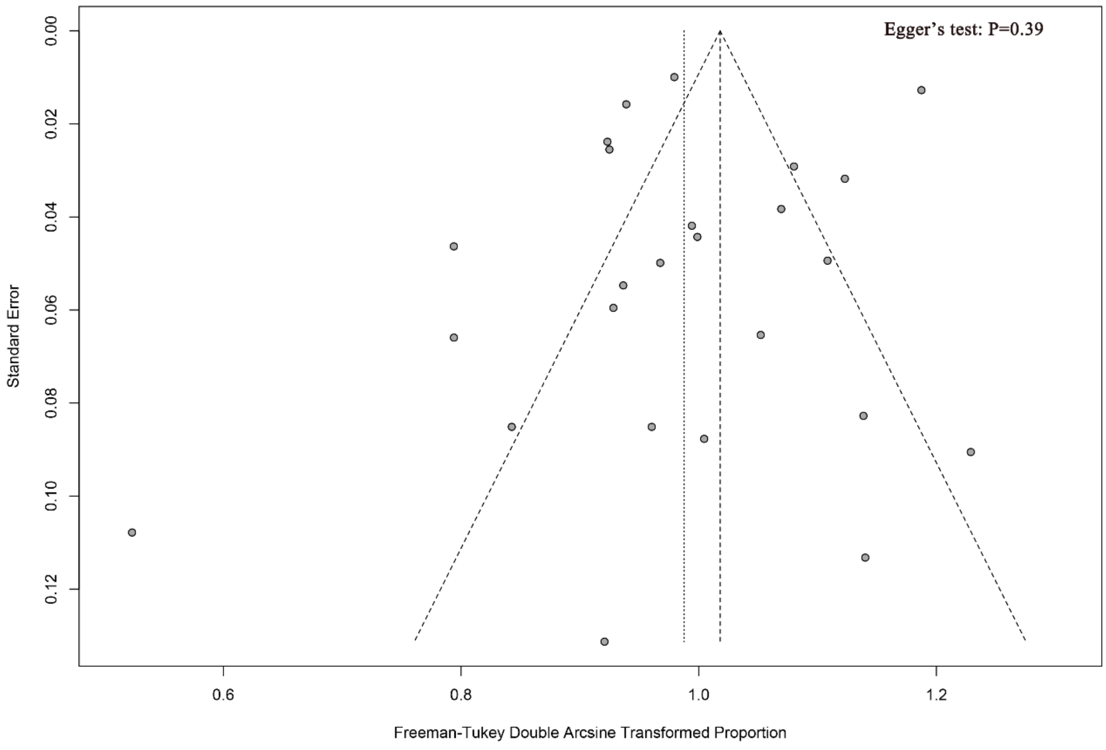

Supplementary Table 1 Sensitivity analysis of ^{68}Ga-PSMA-11 PET/MRI for biochemically recurrent prostate cancer

	^{68}Ga-PSMA-11 PET/MRI	
	Positivity rate (95% CI)	I^2
Omitting Glemser et al.	0.73 [0.63,0.82]	82.40%
Omitting Afshar et al.	0.72 [0.63,0.81]	82.30%
Omitting Grubmüller et al.	0.74 [0.65, 0.82]	81.50%
Omitting Guberina et al.	0.74 [0.64, 0.82]	82.30%
Omitting Mai et al.	0.74 [0.65,0.82]	81.90%
Omitting Joshi et al.	0.73 [0.64,0.82]	82.50%
Omitting T. Lake et al.	0.71 [0.62, 0.79]	78.10%
Omitting Kranzbühler et al.	0.74 [0.66,0.82]	80.00%
Omitting Lütje et al.	0.70 [0.63, 0.76]	71.60%
Omitting Mapelli et al.	0.73 [0.63, 0.82]	82.40%
Omitting Martinez et al.	0.74 [0.66, 0.83]	78.60%
Omitting Alonso et al.	0.73 [0.63, 0.81]	82.40%
Omitting Freitag et al.	0.72 [0.63, 0.81]	81.20%

Supplementary Table 2 Sensitivity analysis of ^{68}Ga-PSMA-11 PET/CT for biochemically recurrent prostate cancer.

	^{68}Ga-PSMA-11 PET/CT	
	Positivity rate (95% CI)	I^2
Omitting Gühne et al. 2022	0.70 [0.65; 0.75]	93.20%
Omitting Duan et al. 2022	0.70 [0.65; 0.75]	93.30%
Omitting Uprimny et al. 2021	0.70 [0.65; 0.75]	92.90%
Omitting Lengana et al. 2021	0.71 [0.67; 0.75]	92.90%
Omitting Plaza López et al. 2021	0.70 [0.65; 0.75]	93.30%
Omitting Yuminaga et al. 2021	0.70 [0.65; 0.75]	93.00%
Omitting Tseng et al. 2021	0.70 [0.65; 0.75]	93.30%
Omitting Strauss et al. 2021	0.70 [0.65; 0.75]	93.30%
Omitting Ribeiro et al. 2021	0.71 [0.66; 0.75]	93.10%
Omitting Morawitz et al. 2021	0.71 [0.65; 0.74]	93.20%
Omitting Lawal et al. 2021	0.69 [0.64; 0.74]	93.10%
Omitting Kroenke et al. 2021	0.70 [0.65; 0.75]	93.30%
Omitting Jentjens et al. 2021	0.70 [0.66; 0.75]	93.20%
Omitting Fourquet et al. 2021	0.70 [0.65; 0.74]	93.20%
Omitting Dadgar et al. 2021	0.70 [0.65; 0.74]	93.30%
Omitting Cerci et al. 2021	0.70 [0.65; 0.75]	92.70%

Omitting Carvalho et al. 2021	0.70 [0.65; 0.75]	93.20%
Omitting Afshar-Oromieh et al. 2021	0.70 [0.65; 0.75]	92.80%
Omitting Seniaray et al. 2020	0.70 [0.65; 0.75]	93.30%
Omitting Regula et al. 2020	0.69 [0.65; 0.74]	93.20%
Omitting Rauscher et al. 2020	0.70 [0.65; 0.74]	93.20%
Omitting Radzina et al. 2020	0.70 [0.65; 0.75]	93.30%
Omitting Miksch et al. 2020	0.71 [0.66; 0.75]	92.80%
Omitting Huits et al. 2020	0.70 [0.65; 0.75]	93.30%
Omitting Abghari-Gerst et al. 2021	0.69 [0.64; 0.74]	81.20%
